# Disturbances of Ligand Potency and Enhanced Degradation of the Human Glycine Receptor at Affected Positions G160 and T162 Originally Identified in Patients Suffering from Hyperekplexia

**DOI:** 10.3389/fnmol.2015.00079

**Published:** 2015-12-22

**Authors:** Sinem Atak, Georg Langlhofer, Natascha Schaefer, Denise Kessler, Heike Meiselbach, Carolyn Delto, Hermann Schindelin, Carmen Villmann

**Affiliations:** ^1^Institute for Clinical Neurobiology, Julius-Maximilians-University of WürzburgWürzburg, Germany; ^2^Bioinformatics Department, Institute of Biochemistry, Friedrich-Alexander-University Erlangen-NürnbergErlangen, Germany; ^3^Rudolf Virchow Center for Experimental BiomedicineWürzburg, Germany

**Keywords:** Cys-loop receptor, glycine receptor, loop B, side chain properties, ligand potencies, hyperekplexia

## Abstract

Ligand-binding of Cys-loop receptors is determined by N-terminal extracellular loop structures from the plus as well as from the minus side of two adjacent subunits in the pentameric receptor complex. An aromatic residue in loop B of the glycine receptor (GlyR) undergoes direct interaction with the incoming ligand via a cation-π interaction. Recently, we showed that mutated residues in loop B identified from human patients suffering from hyperekplexia disturb ligand-binding. Here, we exchanged the affected human residues by amino acids found in related members of the Cys-loop receptor family to determine the effects of side chain volume for ion channel properties. GlyR variants were characterized *in vitro* following transfection into cell lines in order to analyze protein expression, trafficking, degradation and ion channel function. GlyR α1 G160 mutations significantly decrease glycine potency arguing for a positional effect on neighboring aromatic residues and consequently glycine-binding within the ligand-binding pocket. Disturbed glycinergic inhibition due to T162 α1 mutations is an additive effect of affected biogenesis and structural changes within the ligand-binding site. Protein trafficking from the ER toward the ER-Golgi intermediate compartment, the secretory Golgi pathways and finally the cell surface is largely diminished, but still sufficient to deliver ion channels that are functional at least at high glycine concentrations. The majority of T162 mutant protein accumulates in the ER and is delivered to ER-associated proteasomal degradation. Hence, G160 is an important determinant during glycine binding. In contrast, T162 affects primarily receptor biogenesis whereas exchanges in functionality are secondary effects thereof.

## Introduction

Glycine receptors (GlyRs) are heteropentameric ligand-gated ion channels and belong into the superfamily of Cys-loop receptors (Lynch, 2004). Glycinergic disinhibition based on GlyR mutations is associated with neuromotor deficiencies (Schaefer et al., 2013). The immunoglobulin-like structure of the GlyR N-terminus is determined by a short α-helix and 10 β-sheets connected by loop structures forming the large extracellular domain (ECD) followed by four transmembrane domains (TM1-4) and a short C-terminus (Du et al., 2015; Huang et al., 2015; Moraga-Cid et al., 2015). The ECD harbors the agonist and antagonist binding sites formed by loops A, B, C from one subunit and loops E, F, G from an adjacent subunit (Brams et al., 2011; Yu et al., 2014). The inhibitory GlyR complex is formed by three α subunits (α1, α2, α3) and a single β subunit arranged in a 2α:3β configuration (Grudzinska et al., 2005).

Glycinergic inhibition is most important in adult brain stem and spinal cord mediating processes such as motor control, pain sensitization and respiratory rhythm. Concerning the nerve-muscle circuit, GlyRs are postsynaptically expressed in the membrane of motoneurons. Upon glycine-release from neighboring inhibitory interneurons GlyRs get activated and a chloride ion influx leads to hyperpolarization of the motoneurons, balancing excitation, and consequently muscle contraction and relaxation (Rajendra et al., 1997).

Mutations in the GlyR α1 subunit gene *GLRA1* are the most common cause for the rare neuromotor disorder hyperekplexia (Stiff baby syndrome, Startle disease, OMIM 149100). Typical symptoms are neonatal hypertonia and exaggerated startle response observed shortly after birth. Several mutations associated with hyperekplexia have been detected all over the GlyR α1 sequence. Most dominant mutants cluster in TM2 forming the ion channel domain and adjacent loop structures with some exceptions. Recessive mutations are distributed over the entire α1 sequence (Harvey et al., 2008; Schaefer et al., 2013; Bode and Lynch, 2014). A previous classification of dominant mutants affecting channel function and recessive mutants disrupting receptor biogenesis has recently been specified by defective neuronal subcompartimental trafficking. This study concentrated on GlyR loop B (G160R, T162M) and loop D (W68C, D70N, R72H) residues (Schaefer et al., 2015). Translational approaches based on human mutations from patients identified important GlyR residues associated with ligand-binding, conformational changes, ion channel gating, opening, desensitization, and trafficking (Saul et al., 1999; Villmann et al., 2009; Chung et al., 2010; Bode and Lynch, 2013; Bode et al., 2013). The process of ligand-binding is not only mediated by residues within the binding site, but also by the small extracellular loop between TM2-3. The underlying mechanism is thought to involve conformational rearrangements further down in the structure (Maksay et al., 2008; Pless and Lynch, 2009; Lape et al., 2012). Other residues such as P250 localized in loop TM1-2 increase receptor desensitization by altering TM positioning after ion channel opening (Saul et al., 1999; Breitinger et al., 2001). Furthermore, affected arginine residues at the N-terminal end of transmembrane helices (R252 before TM2 and R392 lining TM4) are important start or stop signals for TM helices and therefore most probably interfere with receptor membrane integration. Other residues in recessive hyperekplexia (S231 and I244) localized in TM1 have only marginal effects on receptor biogenesis. The observed decrease in amount of receptor at the cell surface is still sufficient to form functional channels (Vergouwe et al., 1999; Villmann et al., 2009). A recent study on mutations in loop D, which is opposed to the protein surface, showed a largely diminished receptor trafficking due to accumulation of the majority of mutated receptor protein in the ER compartment. Based on a cellular lack of quality control in the ER, receptor subpopulations are able to cycle toward the secretory pathways of Golgi compartments and finally reach the cell surface. The numbers of surface receptors, however, were insufficient to enable functional ion channel formations (Schaefer et al., 2015). Although, the X-ray structure of GlyRs has been recently solved (Du et al., 2015; Huang et al., 2015), domain analysis will help to understand the molecular processes of neurotransmitter binding, translation of ligand-binding into channel opening and receptor closure as well as to describe the pathomechanisms in affected patients.

Here, we analyzed the recently discovered human mutations G160R and T162M in loop B of the ECD and exchanged the affected residues by other amino acids with respect to differences in side chain volume and charge at amino acid positions G160 and T162 close to the glycine-binding site. Our aim was to understand the importance of both residues within the aromatic net shaping the conformation of the neurotransmitter-binding site. We introduced at position G160 an alanine and a serine present in other Cys-loop receptors at the corresponding amino acid position. Both residues, G160 and T162, carry small and uncharged side chains compared to the positively charged arginine and the methionine carrying a hydrophobic side chain identified in hyperekplexia patients. At position T162 we converted threonine into a negatively charged aspartic acid (present in GABA_C_π and AChRα5), an asparagine able to form hydrogen bond interactions with the peptide backbone (similar to ELIC), and proline providing conformational rigidity (present in GABA_A_ γ2). The main focus of this study was on ligand-efficacy and receptor trafficking. Whereas position 160 primarily leads to decreased ligand potency, lowered agonist potency is a secondary effect for mutations at amino acid position 162. Changes at residue 162 located at the C-terminal end of loop B result in ER accumulation with 50% or less receptor leaving the ER toward the cell surface and the formation of functional ion channels with highly disturbed agonist efficacies. Thus, the appropriate sequence organization of loop B is a key component for the conformation of the neurotransmitter binding-pocket of the inhibitory GlyR.

## Materials and methods

### Site-directed mutagenesis

All mutations were introduced by PCR using primers carrying the appropriate mutation at the desired position (Invitrogen, Darmstadt, Germany). Amplimers carrying the mutations were digested with restriction enzymes for cloning (Xho I and Hind III). As parental clone GlyR α1 cDNA in the vector pRK5 was used. After digestion with Xho I and Hind III the PCR products were subcloned into GlyR α1 wild type (wt). All mutations were verified by sequencing (LGC Genomics, Berlin, Germany).

### Cell lines

HEK293 cells (Human embryonic kidney cells) were purchased from ATCC and grown in Earle's minimal essential medium (MEM) supplemented with 10% fetal calf serum, 200 mM GlutaMAX, 100 mM sodium pyruvate and 50 U/mL penicillin/streptomycin (Sigma-Aldrich, St. Louis, MO, USA) under standard growth conditions at 37°C and 5% CO_2_. COS7 cells (African green monkey kidney cells) were purchased from ATCC and grown in Dulbecco's modified Earle's medium (DMEM) with the same supplements added for HEK293 cells and under the same growth conditions.

### Transfection

HEK293 cells were transiently transfected using a modified calcium-phosphate precipitation method, were a mixture of plasmid DNA, CaCl_2_, and 2x HBS buffer (50 mM HEPES, 12 mM glucose, 10 mM KCl, 280 mM NaCl, 1.5 mM Na_2_HPO_4_) was applied onto the cells.

COS7 cells were transfected with DNA diluted in PBS plus 10 mg/mL DEAE-dextran (Sigma-Aldrich, St. Louis, MO, USA). After 30 min of incubation under standard conditions, media was aspirated and fresh media containing 10 mM chloroquine was applied for 3 h. All experiments were done 24–48 h post-transfection.

### Biotinylation of cell surface protein

The biotinylation assay was performed on HEK293 cells transiently expressing the desired GlyR α1 (wt) or α1 variants. Cells were plated on 10 cm dishes. 48 h after transfection, medium was removed and cells were washed three times with ice-cold PBS (GE Healthcare, Freiburg, Germany). The surface proteins were labeled by incubating the cells for 30 min with 1 mg/mL EZ-Link Sulfo-NHS-LC-biotin (sulfonosuccinimidyl-6-(biotin-amido)-hexanoate (Pierce Biotechnologies, Rockford, IL, USA), followed by incubation with quenching buffer (192 mM glycine, 25 mM Tris in PBS, pH 8.0) for 10 min. Cells were detached by using ice-cold PBS buffer followed by centrifugation for 10 min at 1.000 g. Cell lysis was performed with TBS (Tris-buffered saline) with 1% Triton-X100 and protease inhibitor mixture tablet (Roche Diagnostics, Mannheim, Germany) and centrifuged for 1 min at 13.000 g. The supernatant (whole protein fraction) was incubated with 50 μl of streptavidin-agarose beads (Pierce Biotechnologies, Rockford, IL, USA) for 2 h at 4°C while rotating. After removing the supernatant, beads were washed three times in TBS buffer. Biotinylated proteins were eluted by boiling with 50 μl of 2x SDS buffer for 5 min at 95°C. 40 μg of surface proteins were analyzed by Western blot.

### Protein degradation analysis on whole cell lysates

HEK293 cells transiently expressing GlyR α1 wt or α1 variants were incubated under standard growth conditions with proteasome or lysosome inhibitors for specific time periods (all inhibitors have been obtained from Sigma-Aldrich, St. Louis, MO, USA). Cells were incubated with 1 μM of MG132 for 1, 2, and 4 h. The lysosome inhibitor leupeptin (200 μg/mL) was incubated for 6, 12, and 24 h together with the transfected cells.

At each time point, media was aspirated and cells were washed twice with PBS followed by incubation with CytoBuster Protein Extraction Reagent (Merck Millipore, Darmstadt, Germany) supplemented with protease inhibitor for 5 min at room temperature. The resulting cell suspension was centrifuged for 5 min at 16.000 g and 4°C resulting in a supernatant containing solubilized proteins.

*Western Blot Analysis—*For SDS-PAGE, 11% polyacrylamide gels were freshly prepared, followed by Western blot on nitrocellulose membranes (GE Healthcare, Freiburg, Germany). Membranes were blocked for 1 h with 5% BSA in TBS-T (TBS with 1% Tween 20). Primary antibodies were incubated overnight at 4°C. GlyR α1 wt and GlyR mutants were detected with the pan-α antibody MAb4a (Synaptic Systems, Göttingen, Germany), recognizing an epitope in the N-terminus of the GlyRs (residues 96–105). In biotinylation experiments, cadherin was chosen as loading control detected by the pan-Cadherin antibody (Cell Signaling Technology, Danvers, MA, USA). For lysate analysis, GAPDH served as a loading control (Calbiochem, Darmstadt, Germany). Signals were detected using the ECLplus system (GE Healthcare, Freiburg, Germany).

### Immunocytochemical staining

Surface receptors were labeled following a co-transfection of HEK293 cells with GlyR α1 (wt) or α1 variants together with pDsRed-Monomer-Mem. pDsRed-Monomer-Mem (Clontech, Mountain View, CA, USA) encodes a fusion protein consisting of neuromodulin (GAP-43) and a red fluorescent protein used as plasma membrane marker. For surface staining of GlyR α1 in non-permeabilized cells, live cell staining was performed using MAb2b for 1 h at 4°C (1:500 in medium) as primary antibody recognizing a native epitope (residue 1–10 of mature protein at GlyR α1; Synaptic Systems, Göttingen Germany). Intracellular GlyR α1 staining was done following a co-transfection of HEK293 cells of α1 wt or α1 variants and pDsRed-ER. pDsRed-ER (Clontech, Mountain View, CA, USA) harbors the sequence information for a fusion protein of calreticulin and red fluorescent protein and can therefore be used as an ER marker. Cell staining was performed following fixation with 4% paraformaldehyde and 4% sucrose and a permabilisation/blocking step using PBS, 5% goat serum and 0.1% Triton-X100. Here, the primary antibody MAb4a (Synaptic Systems, Göttingen Germany) diluted 1:500 in PBS, 5% goat serum was used able to recognize the denatured epitope. Cells were washed three times with PBS before the secondary antibodies were applied for 30 min. Here, goat anti-mouse Alexa488 or goat anti-mouse Cy3 antibodies (Dianova, Hamburg, Germany) were used 1:500 in PBS, 5% goat serum. After a final wash, cells were embedded in Mowiol containing DAPI (1:20.000) to mark the nucleus.

### Compartmental staining

COS7 cells transiently expressing GlyR α1 (wt) or GlyR α1 variants were stained in permeabilized cells with primary antibodies MAb4a (Synaptic Systems, Göttingen Germany) and a polyclonal anti-calnexin antibody (1:500, Abcam, Cambridge, UK) for ER staining. The detection of ERGIC was done by the monoclonal ERGIC53 antibody (1:500, Enzo Life Science, Lörrach, Germany) and cis-Golgi stainings using a monoclonal antibody anti-GM130 (1:500, BD Transduction Laboratories, Heidelberg, Germany) together with a GlyR α1 specific antibody (Chemicon, Darmstadt, Germany). Secondary antibodies used were goat anti-mouse Cy3/Alexa488 and, goat anti–rabbit Cy3 (Dianova, Hamburg, Germany) diluted 1:500. All stainings were subjected to confocal microscopy on a DMIRE2 confocal microscope.

### Electrophysiology

Maximal current amplifications (I_max_) were measured by the patch clamp technique in a whole-cell configuration mode. Current signals were amplified with an EPC-9 amplifier (HEKA, Lambrecht, Germany). 24 h after transfection, whole cell recordings from HEK293 cells were performed by application of ligand (glycine) in different concentrations using a U-tube system. The extracellular buffer consisted of 137 mM NaCl, 5.4 mM KCl, 1.8 mM CaCl_2_, 1 mM MgCl_2_, 11 mM EGTA, 10 mM HEPES, with a pH adjusted to 7.4 with NaOH. The internal buffer was 120 mM CsCl, 20 mM N(Et)_4_Cl, 1 mM CaCl_2_, 3 mM MgCl_2_, 11 mM EGTA, 10 mM HEPES with a pH adjusted to 7.4 with CsOH. Recording pipettes were fabricated from borosilicate capillaries with an open resistance of about 4 MΩ. Current responses were measured at a holding potential of −60 mV. All experiments were carried out at room temperature. Error bars refer to S.E.M. values.

### Computational methods

The homology model of the GlyR α1 was generated by using the crystal structure of the glutamate-gated chloride channel (GluCl) at 3.35 Å resolution (pdb code: 3RIF) as a template (Hibbs and Gouaux, 2011). Both sequences were aligned according to the ClustalW algorithm using the default settings of the input form found at http://www.ch.embnet.org/software/ClustalW.html (Thompson et al., 1994). The template structure was modified by removing the Fab molecule bound to GluCl. Molecular modeling was performed using MODELLER9.9 (Sánchez and Sali, 2000) with the ligand module. The disulfide bridges were checked in the resulting model. The modeled structure was improved by 200 steps of conjugated gradient energy minimization using the Powell algorithm in Sybyl7.3 2002 St. Louis, MO, USA. The quality of the model was verified by WHAT_CHECK (Hooft et al., 1996). Mutations were introduced and positioned using Coot (Emsley et al., 2010). The visualization of the structures was performed with the PyMOL Molecular Graphics System, Version 1.7.6 (Schrödinger, LLC).

### Statistical analysis

Concentration-response curves were constructed from the peak current amplitudes obtained with at least seven appropriately spaced concentrations in the range 1–10.000 μM glycine. Using a non-linear algorithm (Microcal Origin), concentration-response data were first analyzed using the following Hill equation *I*_glycine_*/l*_sat_ = [*glycine*] ^*n*^^Hill^ / [glycine]^*nHill*^ + EC${}_{50}^{nHill}$ where *I*_glycine_ refers to the current amplitude at a given glycine concentration, *I*_sat_ is the current amplitude at saturating concentrations of glycine, EC_50_ is the glycine concentration producing half-maximal current responses, and *n*_Hill_ is the Hill coefficient.

## Results

### Localization of loop B mutants within the GlyR

**Figure 1:**
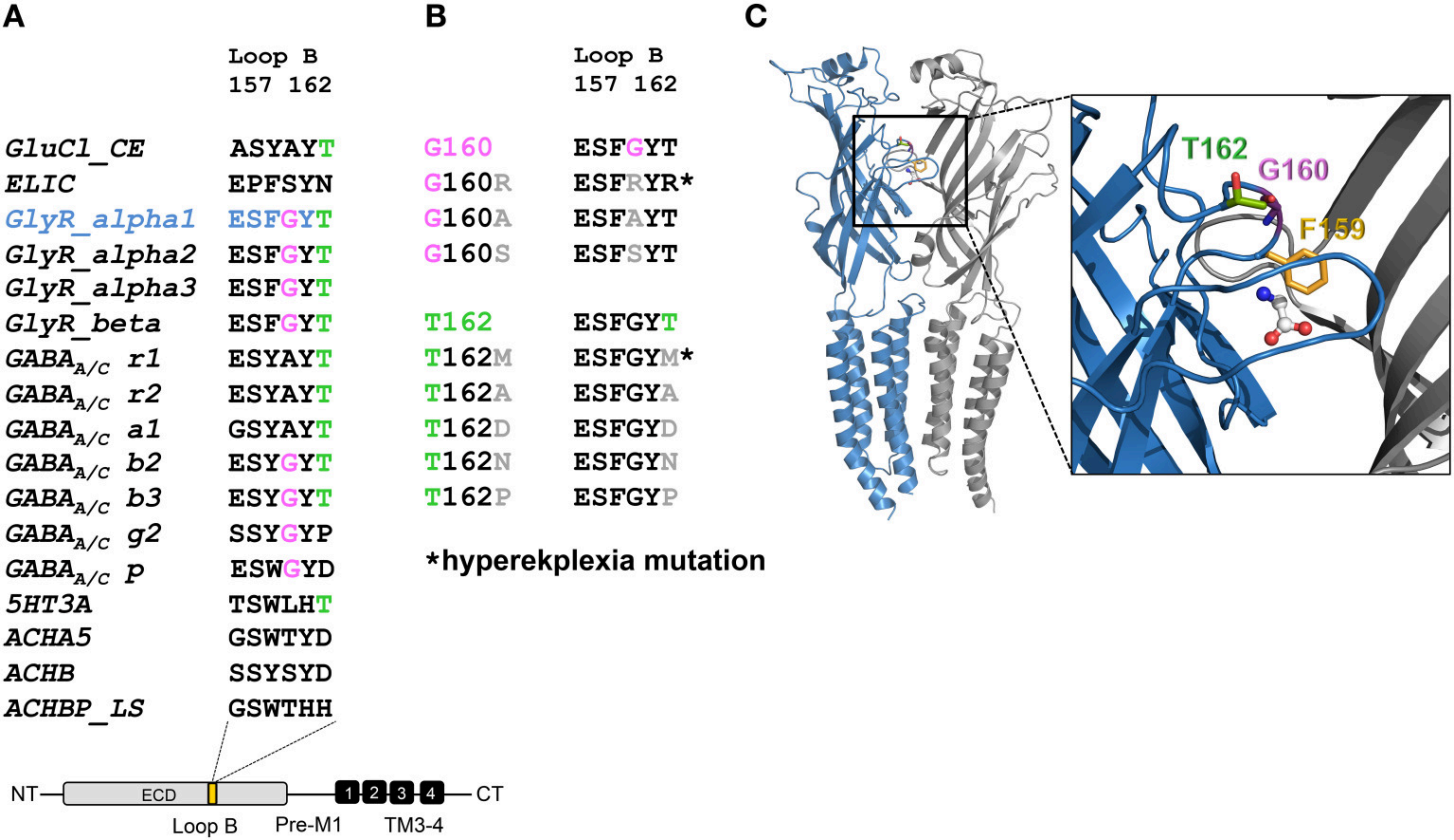
**Overview domain architecture of the GlyR**. **(A)** The loop B region of amino acid residues 157–162 is highly conserved within the family of Cys-Loop receptors. All glycine receptor subunits (α1–blue, α2, α3, β) carry the sequence of ESFGYT. A glycine is localized at position 160 (pink) and a threonine at position 162 (green). **(B)** Loop B mutations (gray letter) are shown and residues linked to hyperekplexia are marked by an asterisk (^*^). Glycine 160 was mutated into an alanine G160A and a serine G160S. Threonine at position 162 was mutated into alanine, aspartate, asparagine and proline (T162A, D, N, and P). **(C)** Representation of two GlyR α1 subunits of a receptor (blue and gray chain, respectively). For this homology model the crystal structure of GluCl (3RIF) was used as a template. Within the loop B region of amino acid 157–162 (enlarged inlet) between β-sheets 7 and 8 of the ECD residues G160 (pink), T162 (green), and F159 (yellow) are marked.

A homology model of the GlyR α1 was generated from the GluCl crystal structure. The region between residues 157 and 162 is conserved within all GlyR subunits (Figure 1A). A recently discovered patient suffering from hyperekplexia carries a mutation at position G160 (pink) where glycine was mutated to arginine (G160R). Another patient mutation is the conversion of T162 (green) to methionine (T162M) again present in loop B of the GlyR α1 ECD. Apart from these human mutations, we generated constructs harboring other amino acids at both positions G160 and T162 corresponding to residues in other Cys loop receptors at the appropriate loop B position (Figures 1A,B). G160A represents the change of a small hydrophobic side chain compared to glycine whereas G160S harbors a hydrophilic side chain. We also created T162A (small hydrophobic side chain), T162D (negatively charged side chain), T162N (hydrophilic side chain), and T162P (sterically demanding side chain). Both residues G160 and T162 are localized in the center of the neurotransmitter-binding site between adjacent subunits and close to the aromatic residue F159, which was suggested to directly interact with the incoming ligand glycine (Figure 1C).

### Impact of residues 160 and 162 in loop B on receptor expression

**Figure 2:**
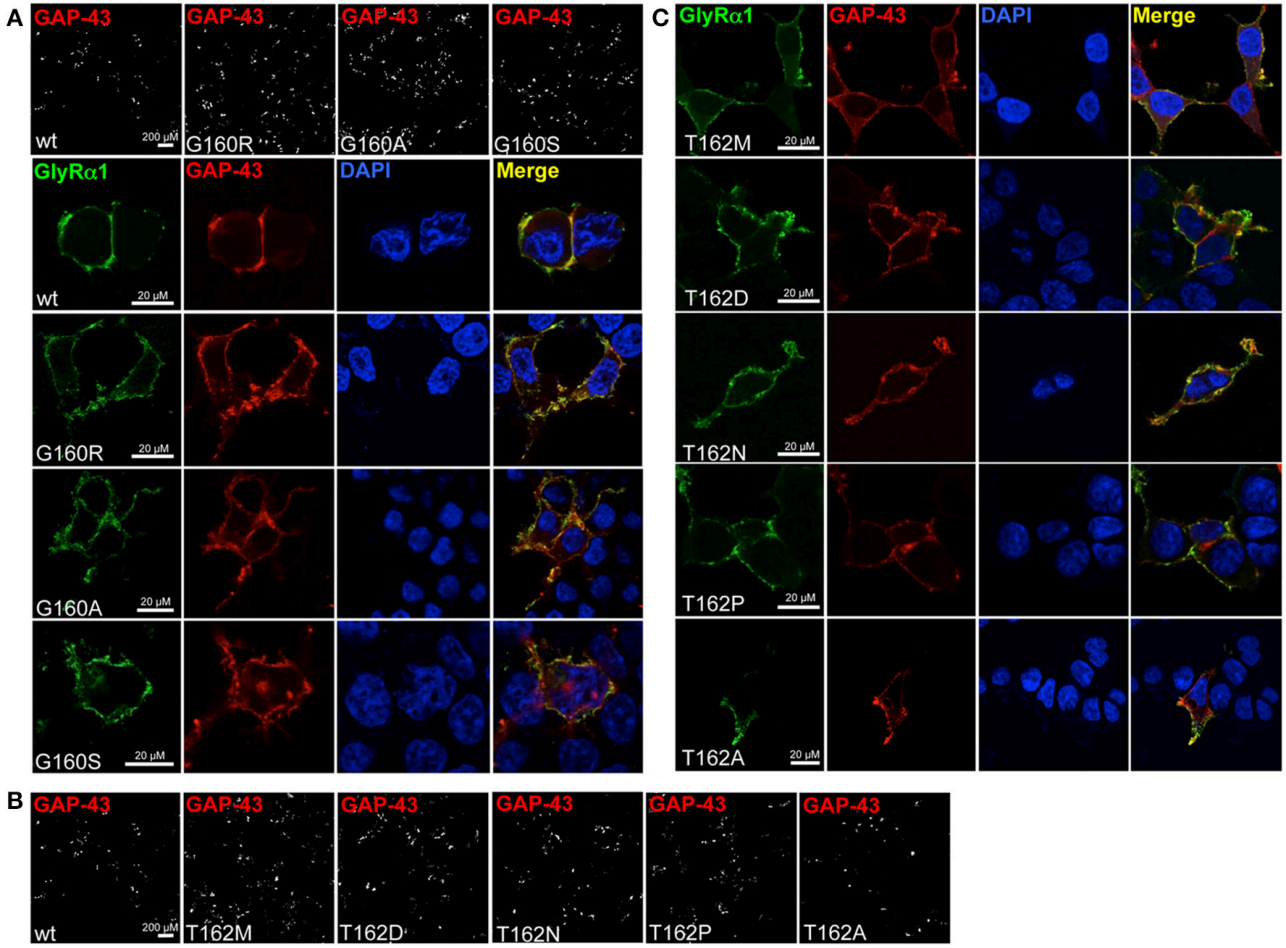
**Integration of GlyR variants into the cellular membrane**. Different α1 mutants were expressed in HEK293 cells following co-transfection of a marker for membrane expression (GAP-43 coupled to dsRed). **(A)** Overview images (first line) represent controls for transfection efficiency (GAP-43) for wt and G160 variants. Below GlyR α1 homomers (green) expressed at cell surface (GAP-43 marker) are shown. G160R, G160A, and G160S mutants are well integrated into the cell surface. **(B)** Transfection efficiency controls for cotransfections of T162 mutants together with GAP-43 coupled to dsRed. **(C)** Mutant GlyRs at position 162 (T162M, T162N, T162D, T162P, and T162A) stained at the cell surface. All GlyR α1 were detected with the monoclonal MAb2b antibody. DAPI was used for staining of the nuclei.

**Figure 3:**
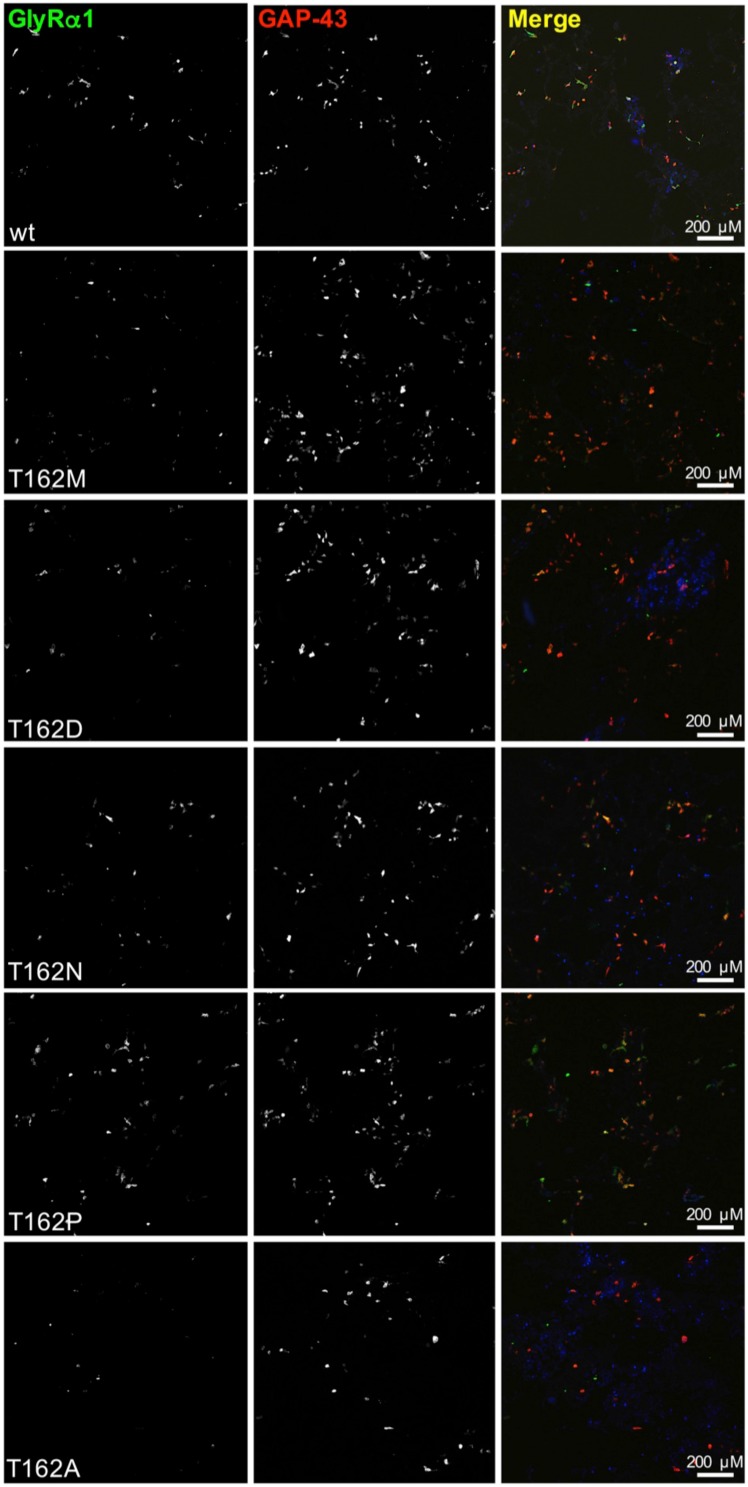
**Overview images demonstrating differences in GlyRα1 expression for T162 mutants**. HEK293 cells were cotransfected with GlyR α1 wt (green) or T162 variants together with the membrane marker GAP-43 coupled to dsRed (red). GAP-43 serves as a marker for transfection efficiency and colocalizes with GlyR α1. Differences in GlyR expression of T162 variants in comparison to α1 wt were observed (first row).

**Figure 4:**
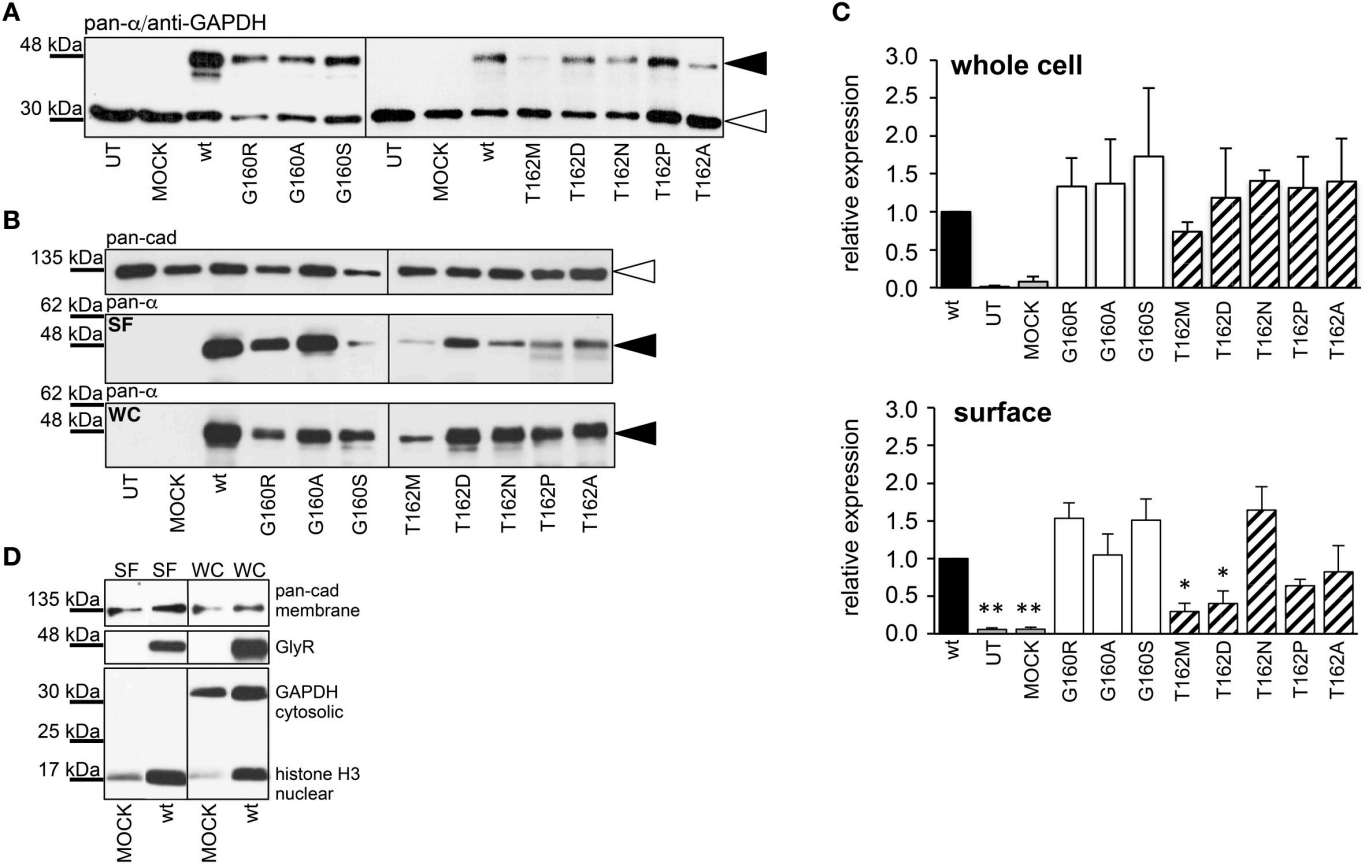
**Quantitative expression levels of mutant GlyRs**. **(A)** Crude HEK293 whole cell lysates transiently expressing GlyR α1 (wt) or α1 variants. Loading control GAPDH 37 kDa. **(B)** Biotinylation pull down experiments to quantify the protein expression of GlyR α1 variants and to distinguish between whole cell (WC) and surface (SF) protein levels. The obtained data were taken from 4 independent experiments. The overall expression seems to be unaffected. Note, differences between surface expression levels of wt and G160 compared to T162 variants. Pan-cadherin was used as a loading control. The GlyR α1 variants were stained at the appropriate molecular weight of 48 kDa (black arrowheads), pan-cadherin appeared at 135 kDa (white arrowheads). **(C)** Quantification of protein levels determined from whole cell (WC) and surface (SF) fractions from 4 independent experiments normalized to pan-cadherin (membrane marker protein). The expression levels are shown in comparison to wild type (wt = 1). Error bars refer to standard error of the mean S.E.M. values. ^*^p < 0.05, ^**^p < 0.01 were considered significant. **(D)** Control for purity of surface protein fraction. Surface (SF) and whole cell (WC) fractions were loaded from MOCK and wt α1 transfected HEK293 cells. Both fractions were stained for the cytosolic protein GAPDH (37 kDa), the membrane marker pan-cadherin (135 kDa) and the nuclear marker histone H3 (17 kDa). Note, histones were stained in all fractions due to (i) their localization attached to the inner nuclear membrane, (ii) naturally occurring posttranslational biotinylation of histones, and (iii) binding to streptavidin beads following spin-down of membranes and solubilization of membrane attached proteins.

Live cell stainings for surface expression showed no obvious differences between GlyR α1 wt and G160 variants (Figure 2A). GAP-43 expressed as a fusion protein with dsRed encoded on a co-transfected plasmid was used as membrane marker and for control of transfection efficiency (Figures 2A,B, 3). The plasma membrane detection of T162 variants at the single cell level did not consider differences to wt (Figure 2C). Although, α1 labeled cells were rare for T162M and T162A (Figure 3). An analysis of crude protein lysates (Figure 4A) was followed by protein quantification from Western blots after pull-down of biotinylated surface proteins by streptavidin binding. Protein lysates provided first evidences for differences in expression levels of α1 wt compared to α1 variants. The biotinylation method does allow a direct comparison between whole cell protein expression and surface protein expression. Here, significant differences of surface protein levels were observed for T162 variants compared to wt α1 (Figures 4B,C). The relative expression of the wt α1 was set to 1 (= 100%). G160 variants were rather unaffected and showed whole cell as well as surface levels comparable to wt. T162M exhibited only marginal affected whole cell expression. All other T162 receptors did not differ in the overall expression levels (Figure 4C). T162M, T162D, T162P, and T162A revealed reduced protein levels at the cellular surface. T162N was the only mutant not affected in trafficking to the cell surface. The mutation T162M identified in a patient with hyperekplexia and T162D demonstrated significantly reduced surface expression levels (T162M 29 ± 10%, T162D 40 ± 16%; Figure 4C). Trafficking of T162P and T162A was decreased but did not reach significance (T162P 64 ± 8%, T162A 82 ± 34%). A determined portion of mutant GlyR protein seems to get stuck on its way to the cell surface most probably in the ER compartment as it has been shown previously for other recessive GlyR α1 mutants (Schaefer et al., 2015). The surface membrane fractions were not contaminated with cytosolic proteins (Figure 4D). Due to centrifugation of all cellular membranes followed by solubilization of proteins out of membranes, all fractions contained histone H3 usually forming tight associations with scaffold proteins of the inner nuclear membrane and are naturally postranslationally biotinylated (Figure 4D; Polioudaki et al., 2001; Bailey et al., 2008).

### Effect of the human mutation T162M on protein trafficking and degradation

**Figure 5:**
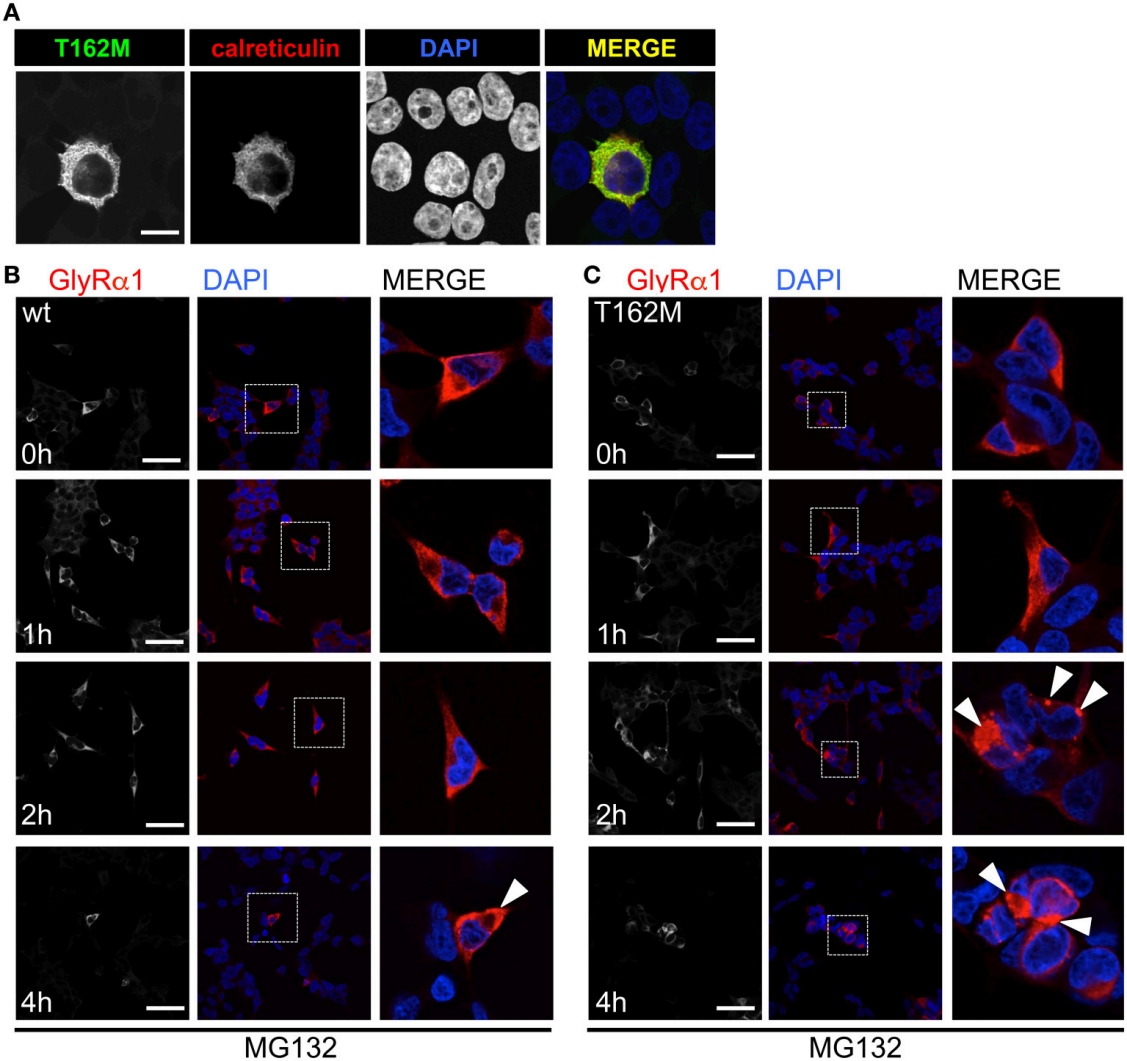
**ER accumulation of T162M**. **(A)** Following cotransfection into HEK293 cells, intracellular stainings of GlyR α1 T162M (MAb4a, green) and the ER marker calreticulin expressed as a fusion protein coupled to dsRed (red). **(B)** Inhibition of the proteasomal pathway using MG132 for 1, 2, and 4 h. Following 2 h presence of MG132, wt transfected cells (MAb4a, red signal) seemed rather unaffected in protein distribution. **(C)** In contrast, T162M (MAb4a, red) showed large accumulations in the cellular lumen (white arrowheads, third lane, right picture). Lower lane represents pictures after 4 h treatment with MG132. Enlarged images are shown in right column. GlyR wt **(B)** signal is dense, whereas T162M **(C)** is characterized by large protein aggregations localized close to the nucleus.

**Figure 6:**
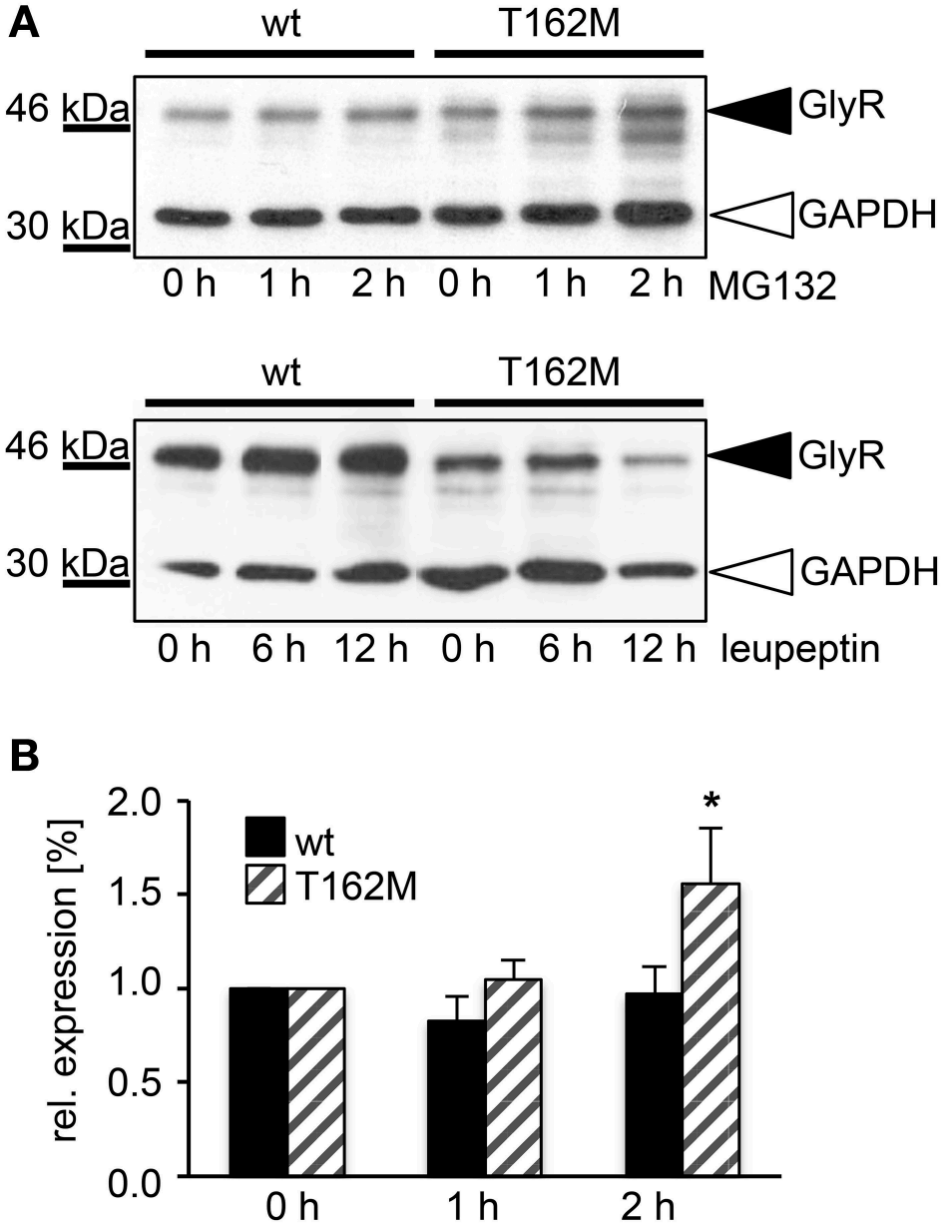
**T162M degradation via the proteasomal degradation pathway**. **(A)** MG132 was added to transfected HEK293 cells expressing either wt or T162M for 0, 1, and 2 h. Following incubation with the proteasomal blocker, cell lysates (40 μg per lane) were analyzed by Western blotting (upper blot) using the GlyR antibody MAb4a (black arrowhead, 48 kDa). GAPDH 37 kDa served as a loading control (white arrowhead). Inhibition of cellular lysosomes by leupeptin for 0, 6, and 12 h (lower blot), GAPDH 37 kDa. GlyR protein is marked by black arrowhead. **(B)** Quantification of protein levels after MG132 incubation for 1 and 2 h from 4 independent experiments. T162M increased after 2 h proteasomal block, ^*^p < 0.05.

T162M colocalizes with calreticulin, a chaperone present in the ER and important for protein quality control (Figure 5A). It has been shown for the nicotinic acetylcholine receptor, that calreticulin together with calnexin interact with newly synthesized glycoproteins in the ER, stabilizing the receptors and assisting in the folding process (Wanamaker and Green, 2007). Misfolded or unassembled protein is degraded via the ER-associated degradation (ERAD) pathways, e.g., via the proteasome. To determine ERAD of mutated GlyR α1, we blocked the proteasomal degradation with the proteasome inhibitor MG132 for different time periods (1, 2, and 4 h). We expected to observe protein accumulation since misfolded receptor proteins would normally be degraded via the proteasomal pathway. After a 1 h treatment with MG132, protein distribution of GlyR α1 wt and T162M was similar in the cytoplasm of transfected cells (Figures 5B,C). The protein expression pattern of wt was rather stable up to 2 h of treatment with only slight densities close to the nucleus at 4 h presence of the blocking agent (Figure 5B). In contrast, accumulation of T162M was already present after 2 h and even more prominent after 4 h of treatment (Figure 5C). MG132 is a highly potent, cell-permeable proteasome inhibitor. Since long incubation with MG132 is toxic to cells, we quantified the GlyR α1 wt and T162M protein amounts following proteasomal blocking following a 2 h presence of MG132 demonstrating a significant increase of T162M protein compared to wt protein (Figures 6A,B). Following 1 h incubation with MG132, the T162M starts to accumulate, which is more prominent at 2 h MG132 treatment. The lower degradation bands do also increase in intensity between 1 and 2 h presence of the blocking agent (Figure 6A). This might argue for less stable protein and higher turnover rates of the mutated GlyRs. The GlyR α1 wt protein is not affected at its protein level by a 2 h treatment with MG132. It has been shown that ubiquitinated GlyRs expressed at the cell surface are degraded by subsequent internalization and lysosomal pathways (Büttner et al., 2001). To test if the mutant T162M also uses this pathway for degradation, leupeptin was utilized to block lysosomal degradation. Following treatment with leupeptin, the T162M mutation did not show any differences upon leupeptin treatment after 6 h but reduced protein levels after a 12 h treatment with leupeptin in contrast to GlyR α1 wt (Figure 6A). The mutant T162M is still degraded in the presence of leupeptin arguing for ongoing degradation using pathways independent of degradation via lysosomes compared to α1 wt.

**Figure 7:**
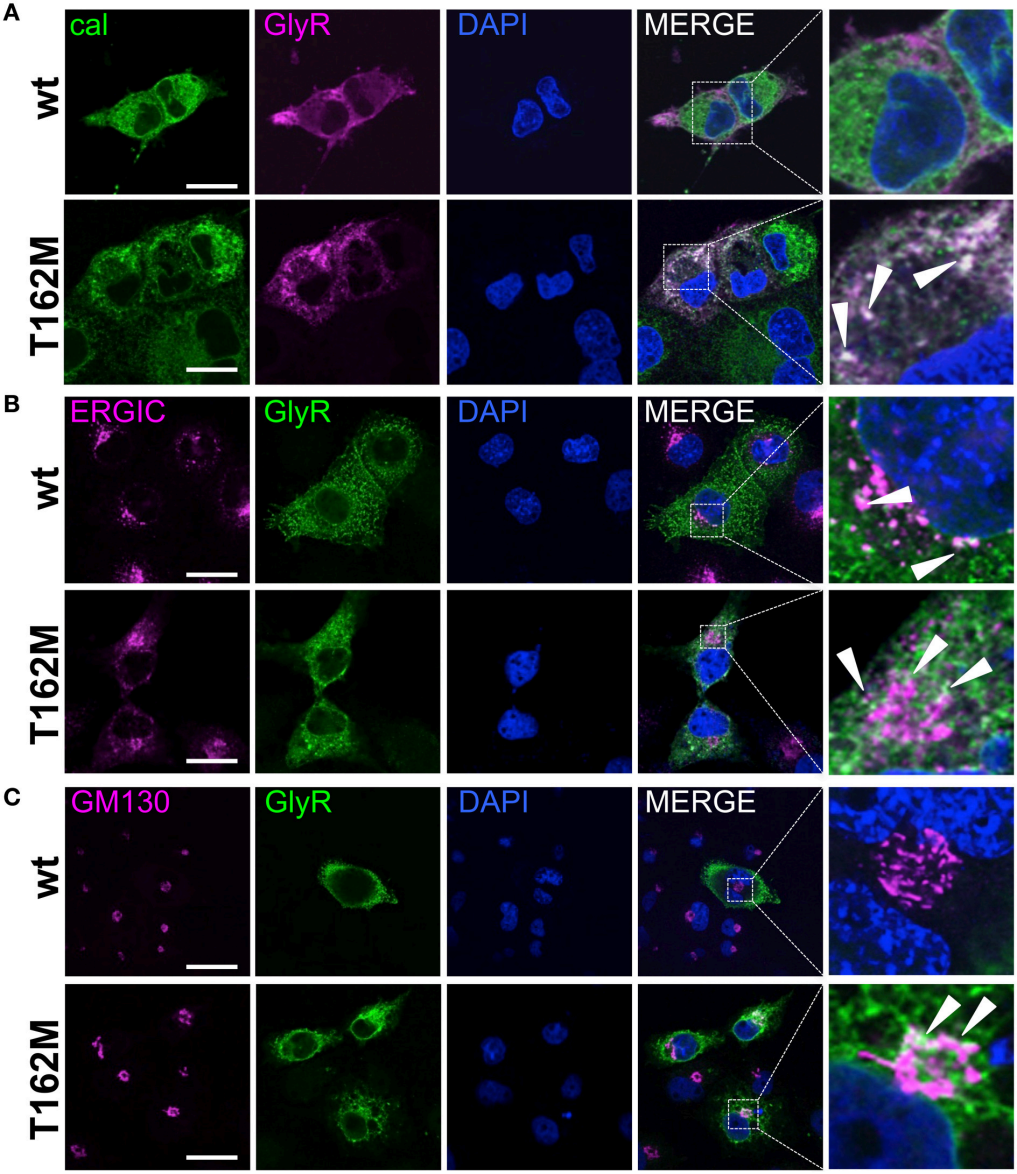
**Trafficking routes of T162M**. **(A)** Costaining of T162M (MAb4a, red) and calreticulin (green), a chaperone and ER quality control protein in the ER in transfected Cos7 cells. Note again large T162 accumulations in the ER compared to wt (upper lane, white dots marked by white arrowheads). **(B)** ERGIC co-staining of GlyR wt and T162M (anti-α1, green) together with ERGIC-53 (pink). **(C)** Cis-Golgi staining using GM130 (pink) as a marker of the secretory Golgi compartment. Mutant GlyR T162M (anti-α1, green) was visible in the cis-Golgi compartment. The white bar represents 30 μm. Right column represents enlargements of merged images (white dotted boxes).

Subcompartmental analysis of GlyR α1 wt and T162M was performed for the compartments ER, ERGIC and cis-Golgi in Cos7 cells using co-stainings with marker proteins present in these compartments. Cos-7 cells have been used for subcompartmental analysis due to their large cytoplasm in comparison to HEK293 cells. Calreticulin, a chaperone localized at the ER exit sites for folded proteins, co-localized with the T162M but only marginally with GlyR α1 wt (Figure 7A). The GlyR α1 wt instead is observed at the outer most cytoplasm and the membrane. T162M forms large accumulations in the ER. ERGIC-53 is a protein of the ER-Golgi intermediate compartment. Subpopulations of T162M pass the ERGIC and cycle toward the cis-Golgi compartment where co-localisation was observed with GM130 (Figures 7B,C). The GlyR α1 wt was only present at some locations in ERGIC but no wt protein was detectable in cis-Golgi, arguing for a fast forward trafficking of the GlyR α1 wt receptors. The large ER accumulations of mutated T162M protein in the ER might not only result from the recognition of abnormally folded or incompletely assembled T162M protein. Retrograde transport mechanisms from cis-Golgi and ERGIC might also underlie this phenomenon.

### Changes in ligand potencies of GlyR α1 loop B variants

**Figure 8:**
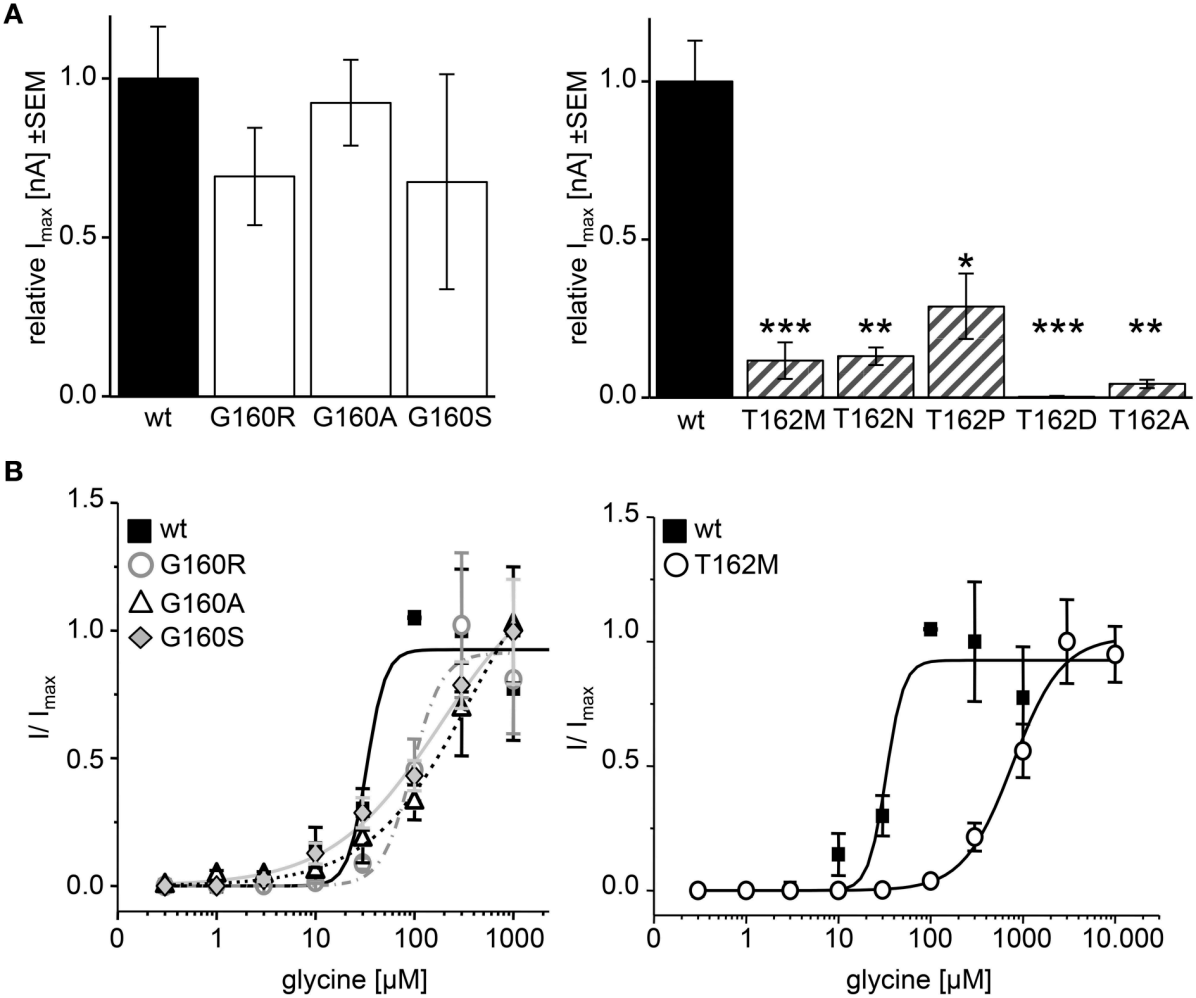
**Physiological changes in agonist potency due to affected loop B residues**. Whole cell recordings on HEK293 cells transiently expressing GlyR α1 or mutant GlyR were performed to analyze receptor functionality and ligand potency. **(A)** Relative maximal currents (I_max_) of G160 variants (left graph, white bars) determined at 100 μM glycine. Note, all T162 variants show reduced maximal currents (right graph, gray striped bars). n, number of independent measurements; *n* = 7; ^*^p < 0.05, ^**^p < 0.01, ^***^p < 0.001. **(B)** EC_50_ measurements using glycine concentrations of 1, 3, 10, 30, 100, 300, 1000, 3000, and 10.000 μM. GlyR α1 wt–black square, solid line; G160R–circles, dot-dashed line; G160A–triangle, dashed line; G160S–diamond, gray line; T162M–white circles, solid line. 1 mM glycine was used as a standard glycine concentration to determine I/Imax. *n* = 5–6.

The physiological characterization of the mutant receptor populations was done by electrophysiological measurements using whole-cell recordings following expression in HEK293 cells. In HEK293 cells, the EC_50_ of glycine for GlyR α1 wt has been reported between 15 and 60 μM. Therefore, we used a glycine concentration of 100 μM to determine whole-cell currents resulting in maximal currents for the wt expressing cells. I_rel_ currents of G160 mutants (white bars, Figure 8A) were similar to wt. G160R evoked 69 ± 10% of the wt current, G160A evoked 92 ± 12% of wt and G160S ended up with 68 ± 22% of wt current. The reduction of the observed inward currents for G160 mutant was, however, not significant (Figure 8A, Table 1). T162 α1 variants (gray striped bars, Figure 8A) showed significantly decreased inward currents at 100 μM glycine. T162M could only achieve 11 ± 5% of I_100μ*M*_ compared to wt. T162N achieved 13 ± 3% and T162P 29 ± 10%. T162A was able to reach 4 ± 1% of maximum current of wt (Figure 8A). Except T162D, all variants were able to form functional receptors but evoked less inward currents. A reduction of the inward current might be due to the observed low expression of these mutated receptor complexes at the cellular surface. The reduction of surface expression might not be the major reason for inward current reduction as demonstrated by T162N, which is indistinguishable from wt in cell surface receptor numbers but resulted in a reduction of 85% of the wt response. For T162D less than 1% of wt currents were measured in two out of five cells recorded arguing for a non-functional mutation.

**Table 1:** **Functional properties obtained from GlyR α1 mutants at positions 160 and 162**.

**Clone**	**Number of independent experiments**	**Expression at cell surface**	**Whole cell expression**	**Number of cells**	**I_max_ [pA] ± SEM**	**Number of cells**	**EC_50_ gly [μM] ± SEM**
GlyR α1 wt	4	+++	+++	7	1571 ± 260	5	36 ± 10
UT	4	-	-	-	-	-	-
MOCK	4	-	-	-	-	-	-
α1 G160R	4	++	+++	7	1089 ± 167	5	92 ± 13
α1 G160A	4	++	+++	8	1453 ± 197	6	≥383
α1 G160S	4	++	+++	8	1061 ± 359	5	≥184
GlyR α1 wt	4	+++	+++	17	2916 ± 371	5	36 ± 10
α1 T162M	4	+	++	10	309 ± 154^***^	6	776 ± 96
α1 T162A	4	++	+++	5	148 ± 45^**^	-	n.d.
α1 T162D	4	+	+++	7	10 ± 6^***^	-	n.d.
α1 T162N	4	+++	+++	5	447 ± 96^**^	-	n.d.
α1 T162P	4	++	+++	5	832 ± 300^*^	-	n.d.

^*^UT refers to untransfected cells and MOCK to GFP transfected cells used as controls. n, number of independent experiments; SEM (standard error of the mean), n.d. not determined, p-values refer to the appropriate WT

* *p < 0.05*,

** *p < 0.01*,

*** *p < 0.001*.

Application of 100 μM glycine did not result in obvious changes for G160 variants. In addition, trafficking of G160 mutants is not affected. Determination of glycine potency however showed significant differences. Using 7 different glycine concentrations in a range of 1–1.000 μM glycine, all G160 mutants showed reduced ligand potencies (EC_50_). GlyR α1 wt achieved its half maximal current at a concentration of 36 ± 10 μM. The EC_50_ value determined for G160R was 92 ± 13 μM. A maximal concentration of glycine (1 mM) was not sufficient to reach saturation for GlyRα variants G160A and G160S. However, both variants exhibit largely decreased agonist potencies with EC_50_ values ≥383 μM for G160A and ≥184 μM for G160S (Figure 8B). The glycine concentration to activate 50% of maximal currents (I_max_) was increased by a factor of 3–10 for G160 mutants. The effect was even more prominent for T162M with more than a 21fold increase in glycine EC_50_. The EC_50_ for the mutant T162M was 776 ± 96 μM (Figure 8B).

### Conformational changes by an introduction of a negatively charged residue in loop B

**Figure 9:**
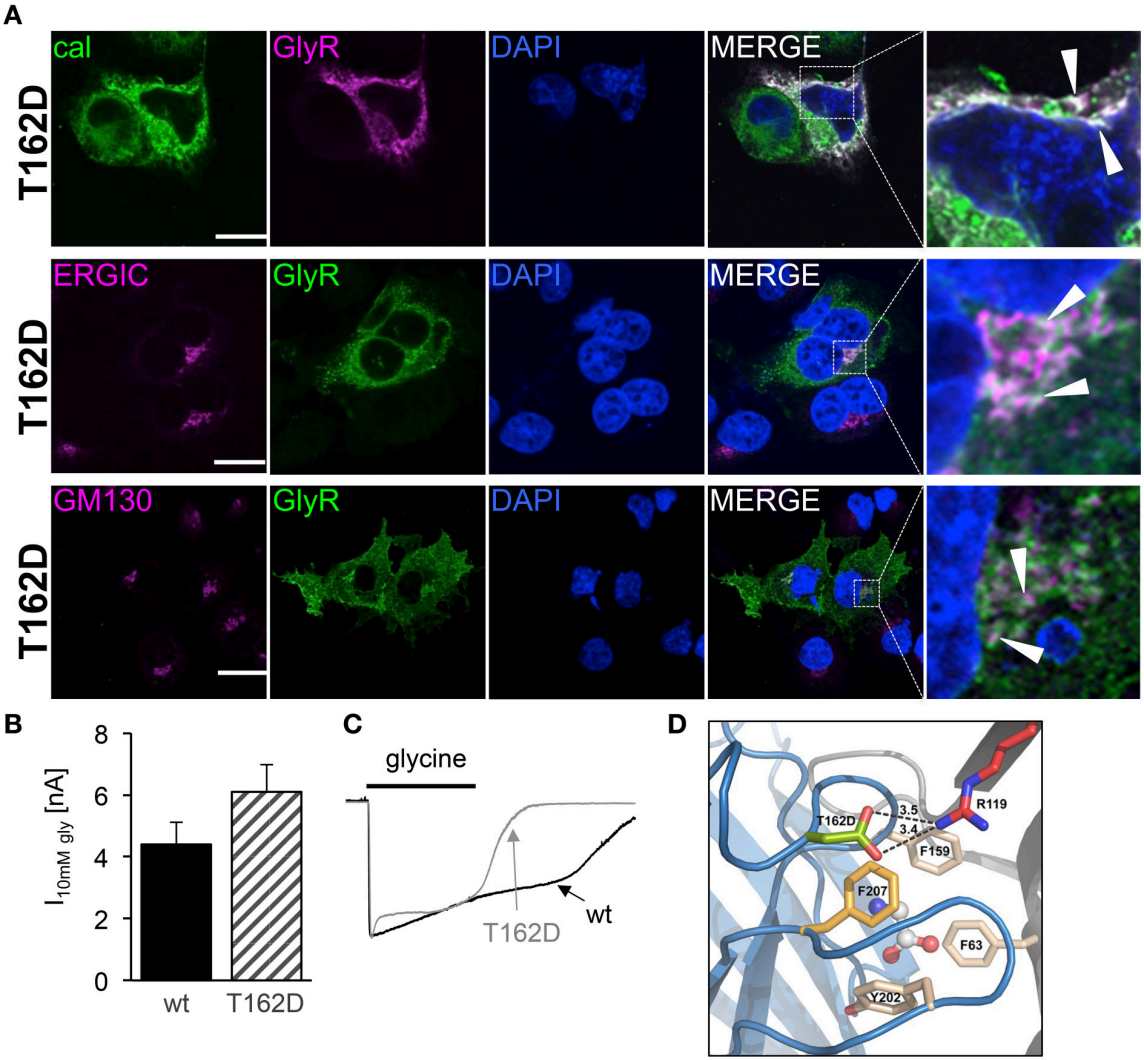
**Subcompartmental distribution of T162D**. **(A)** Transfected Cos7 cells were used to co-stain GlyR α1 T162D (pink) with calreticulin (green). Large ER accumulations (white dots marked by white arrow heads) were obvious (right picture, first lane). Costaining of T162D (anti-α1, green) with ERGIC (pink, second lane), colocalization with GM130 (pink, third lane). Right pictures demonstrate enlarged areas of the white dotted box in the merged picture. **(B)** Whole cell maximal currents of T162D compared to α1 wt at saturating concentrations of glycine (10 mM). **(C)** Single traces of 10 mM recordings from wt and T162D. Note, the fast channel closure compared to wt channels during washout of very high glycine concentrations. **(D)** View from the side onto the ligand-binding site at the interface of two adjacent GlyR subunits (blue and gray), with bound glycine (in ball-and-stick representation). Residue D162 is marked in green, F159 in beige, further aromatic residues lining the glycine binding site (Y202, F63) are beige and R119 shown in red with putative salt bridge indicated by black dashed lines and distances given in Å. Note: F63 and R119 are localized at the neighboring subunit.

Since the mutant T162D did not result in functional ion channels although present to some extent at the cellular surface, we followed T162D trafficking in transfected Cos7 cells (Figure 9A). T162D passed the ERGIC and the cis-Golgi with no obvious changes compared to T162M (Figure 7A). Similar to T162M, ER accumulation was observed. The position T162 in the GlyR seems to influence protein folding followed by ER export (Figure 9A). When high saturating concentrations of glycine (10 mM) were applied to cells expressing T162D, the receptors responded with maximal currents indistinguishable from GlyR α1 wt (Figure 9B). Hence, the mutant T162D did not result in non-functional ion channels rather physiological concentrations of glycine are not sufficient to activate these GlyR channels. Interestingly, the T162D channels close faster during ligand washout compared to wt channels (Figure 9C). Homology modeling of the T162D mutation revealed that the introduction of a negatively charged aspartate likely allows a salt bridge formation with the positively charged arginine R119 present at the neighboring GlyR subunit in the pentameric receptor complex (Figure 9D). Thus, a primary change, putatively in the fold and flexibility of the ligand-binding site due to salt-bridge formation, less ER export and integration in the outer cellular membrane resulted secondary in an ion channel with an unfavorable conformation of the ligand-binding pocket.

## Discussion

Glycinergic disinhibition results from either disrupting the functionality of glycine receptors or trafficking disabilities. So far, mutations in the gene *GLRA1*, the major common cause for the neuromotor disorder hyperkplexia associated with disturbances in glycinergic neurotransmission, have been categorized into dominant mutations associated with functional disruptions and recessive mutants affecting receptor biogenesis (Chung et al., 2010; Bode and Lynch, 2014). Recently, we could demonstrate that disrupted trafficking does not necessarily result in ER accumulation and degradation. Rather, subpopulations of receptor are able to circumvent the ER quality control and traffic through the secretory pathways of the Golgi compartment. However only a minority is able to integrate into the outer membrane. The receptor numbers at the cell surface are insufficient to enable a normal glycinergic function (Schaefer et al., 2015). Here, we analyzed in detail human mutations localized within the GlyR ligand-binding sites resulting from different modes of inheritance dominant—G160R and recessive—T162M.

To get deeper insights into the importance of residues within the ligand-binding site we mutated the affected residues into amino acids present in other subtypes of the Cys-loop receptor family—the closely related inhibitory receptors of the GABA_A∕C_ family, the prokaryotic receptor ELIC and the *C.elegans* receptor GluCl harboring the highest sequence identity to the glycine receptor family among all Cys-loop receptor members (Hilf and Dutzler, 2009; Hibbs and Gouaux, 2011). The mutant G160R as well as the other two mutants G160A and G160S showed no differences in expression levels compared to the wt receptor α1. This was demonstrated by independent methods of immunocytochemical as well as proteinbiochemical analyses. Originally, G160R was identified in a patient with a dominant mode of inheritance (Schaefer et al., 2015). G160 is localized in direct neighborhood to F159, which has been shown to be important for a cation-π interaction with the incoming ligand glycine (Schmieden et al., 1993; Pless et al., 2008). In a recent study using a series of unnatural amino acids, a cation-π interaction between F159 and the amino groups of β-alanine and taurine was also determined. Compared to the interaction with glycine, the strength of interactions with taurine and β-alanine was significantly weaker. This might be due to a different orientation of the partial agonists within the ligand-binding pocket (Pless et al., 2011). Similarly, mutations of other aromatic residues contributing to the ligand-binding interface, e.g., F63, F207 glycine concentrations of up to 300 mM failed to saturate the current responses (Grudzinska et al., 2005). Our analysis of the ligand-binding potencies revealed a 3–10-fold decrease in ligand-efficacy of glycine demonstrating the importance of the small G160 to enable the correct conformation of the ligand-binding pocket for the natural agonist. Interestingly, the positively charged side chain of the arginine did not result in a more drastically affected ligand potency compared to a small side chain change e.g., in G160A. We conclude that the smallest amino acid glycine harboring a hydrogen instead of a side chain at position 160 of the human GlyR α1 is essential to provide the optimal ligand-binding conformation for the neurotransmitter glycine. Nevertheless, we cannot exclude other factors such as differences in the transduction pathways following ligand-binding enabling channel opening.

Residue T162 is also localized within the binding site. The underlying mechanism of the human mutant T162M seems to be a combination of affected biogenesis and functional disruption. Only 50% of T162M receptors are able to reach the cell surface obtained following transfection into heterologous expression systems. Although, ER accumulation was observed, the mutant was also detectable in compartments such as the ER-Golgi intermediate compartment ERGIC as well as the cis-Golgi. Therefore, the overall protein fold of T162M and T162D, P, and A does not lead to a misfold resulting in lack of ER release (Lippincott-Schwartz et al., 2000; Hebert and Molinari, 2007). The subpopulations of T162 mutants reaching the cell surface are, however, not sufficient to enable functionality of the GlyR complex. We hypothesize retrograde signaling of mutated α1 subunits initialized by so far unknown control proteins within the ERGIC and the cis-Golgi compartment resulting in proteasomal degradation (ERAD degradation). Proteasomal degradation has been shown for other recessive mutants (Villmann et al., 2009). Moreover, a higher turnover of mutated α1 receptor was exhibited by pulse-chase experiments. Here, we could demonstrate that the mutated T162M resulted in cytoplasmic accumulation after a 2 h treatment with the proteasomal blocker MG132 in comparison to wt. For the wt receptor, accumulation was first observed after a 4 h treatment but with much less local protein close to the nuclear area compared to T162M. Such accumulations in aggrosome-like structures close to the nucleus have been demonstrated following proteasomal blocking. Aggrosomes assemble to process misfolded proteins that cannot well be handled by the ubiquitin-proteasome pathway (Goldberg, 2003). Our calculation of T162M protein following a block of proteasomal degradation resulted in a significant increase of T162M protein levels after 2 h presence of MG132.

Endocytosis followed by lysosomal degradation has been shown for membrane-associated GlyR α1 wt (Büttner et al., 2001). The incorporation of membrane proteins into endosomal membranes is a common pathway for membrane-bound receptor proteins. The GlyR α1 is a stable glycoprotein with a half-life of approximately 2 days (Hoch et al., 1989). Following a 12 h presence of the lysosomal blocker leupeptin, we indeed could demonstrate accumulations of wt GlyR α1. Therefore, lysosomal degradation does indeed represent the major degradation pathway for the GlyR α1 wt protein.

In contrast, the T162M mutant was observed at much lower receptor protein levels arguing for ongoing degradation in the presence of leupeptin using pathways other than the lysosomal pathway. However, we cannot exclude that the fraction of T162M cell surface receptors might use lysosomal pathways for degradation. The proportion of T162M receptors that does not traffick to the cell surface is degraded via the proteasomal pathway.

The fraction of T162 mutant receptor at the cell surface diminishes glycinergic function to only 20% of wt activity at a glycine concentration of 3-fold above EC_50_ for wt α1. At glycine concentrations above physiological levels 3–10 mM the functionality of T162 mutants was indistinguishable from wt receptors. Concentrations of up to 3.5 mM glycine can be reached during activation (Beato, 2008), however, the fraction of cell surface receptors might still be too low to enable inhibitory neurotransmission in the human organism. The EC_50_ for T162M shows a 21-fold increase indicating a disruption of the ligand-binding site. The artificial mutant T162D resulted in non-functional channels following glycine application of concentrations 3-fold above EC_50_ for wt α1. At very high concentrations of glycine, mutated T162D receptors were able to open. A conformational change required to open the ion channel pore seems therefore not be hindered by the mutation rather high glycine concentrations are necessary to allow activation of channels.

Modeling of the mutant T162D demonstrated that the mutation of T162 into an aspartate might result in novel interactions with other residues from the neighboring subunit. The introduction of the negatively charged aspartate would enable the formation of a salt bridge together with R119 from the neighboring subunit. One might speculate that the novel salt bridge stabilizes the protein conformation and does not result in misfolding allowing receptor forward trafficking from the ER to further cell compartments. Following integration into the cell surface the receptor configuration is less favorable to get activated by the neurotransmitter glycine. *In vivo*, such GlyR α1 mutants may provide cell stress to motoneurons resulting in a more severe phenotype of these patients compared to patients carrying a NULL-allele (Brune et al., 1996; Becker et al., 2006) suffering from a rather mild form of hyperekplexia.

In summary, residues G160 and T162 localized in loop B of the GlyR α1 protein lining the ligand-binding pocket are essential determinants for ligand potency. Both residues when mutated lead to significant decreases in ligand potency. In contrast to G160, where the functional disturbances are a primary consequence of the mutation, the observed functional impairment of T162 mutants is most probably a secondary effect. Primarily, lack of threonine 162 leads to changes in the overall protein biogenesis, but mutant receptors are still sufficient to circumvent the ER quality control. The small fraction of cell surface receptors is not able to sustain normal glycinergic neurotransmission. Hence, loop B residues are crucial elements within the conformation of the ligand-binding pocket.

## Author contributions

Participated in Research design—CV. Conducted experiments—CV, SA, NS, GL, and DK. Performed data analysis—HM, CD, HS, CV, SA, GL, NS, and DK. Wrote the manuscript—CV, SA.

## Funding

This work was supported by DFG VI586 (CV) NS and GL are supported by the GSLS Würzburg.

### Conflict of interest statement

The authors declare that the research was conducted in the absence of any commercial or financial relationships that could be construed as a potential conflict of interest.

## Abbreviations

GlyR: glycine receptor
wt: wild type
ECD: extracellular domain
TM: transmembrane
ER: Endoplasmic reticulum
Cys: cysteine.
